# Prevalence of Y-chromosome sequences and gonadoblastoma in Turner syndrome

**DOI:** 10.1016/j.rppede.2015.12.004

**Published:** 2016

**Authors:** Alessandra Bernadete Trovó de Marqui, Roseane Lopes da Silva-Grecco, Marly Aparecida Spadotto Balarin

**Affiliations:** Universidade Federal do Triângulo Mineiro (UFTM), Uberaba, MG, Brazil

**Keywords:** Turner syndrome, Y chromosome, Gonadoblastoma, Prevalence, Polymerase chain reaction, Gonadal dysgenesis

## Abstract

**Objective:**

To assess the prevalence of Y-chromosome sequences and gonadoblastoma in patients with Turner syndrome (TS) using molecular techniques.

**Data source:**

A literature search was performed in Pubmed, limiting the period of time to the years 2005–2014 and using the descriptors: TS and Y sequences (n=26), and TS and Y-chromosome material (n=27). The inclusion criteria were: articles directly related to the subject and published in English or Portuguese. Articles which did not meet these criteria and review articles were excluded. After applying these criteria, 14 papers were left.

**Data synthesis:**

The main results regarding the prevalence of Y-chromosome sequences in TS were: (1) about 60% of the studies were conducted by Brazilian researchers; (2) the prevalence varied from 4.6 to 60%; (3) the most frequently investigated genes were *SRY, DYZ3* and *TSPY*; (4) seven studies used only polymerase chain reaction, while in the remaining seven it was associated with FISH. Nine of the 14 studies reported gonadectomy and gonadoblastoma. The highest prevalence of gonadoblastoma (33%) was found in two studies. In five out of the nine papers evaluated the prevalence of gonadoblastoma was 10–25%; in two of them it was zero.

**Conclusions:**

According to these data, molecular analysis to detect Y-chromosome sequences in TS patients is indicated, regardless of their karyotype. In patients who test positive for these sequences, gonadoblastoma needs to be investigated.

## Introduction

Turner syndrome (TS) is a chromosomal disorder with an incidence of 1:2500 girls; its etiology is associated with total or partial X-chromosome monosomy and the diagnosis is made by karyotype testing.^1^^,^ 2 A retrospective study of 260 patients with TS showed that the improvement in chromosomal analysis provided a change in the proportion of observed karyotype types.^3^

Patients with TS exhibit have short stature and gonadal dysgenesis as main clinical signs. They also may have low hairline at the nape of the neck, strabismus, ptosis, high-arched palate, micrognathia, short and/or webbed neck, lymphedema of hands and/or feet, metacarpal and/or metatarsal shortening, Madelung deformity, *cubitus valgus, genu valgum*, scoliosis and multiple pigmented nevi, cardiovascular and renal disorders, thyroid disorders, hearing impairment, hypertension, osteoporosis, and obesity.^1^^,^ 2 However, this syndrome is characterized by wide phenotypic variability, from patients with the classic phenotype to those almost indistinguishable from the general population.

Women with TS who have Y chromosome material are at increased risk of developing gonadal tumors, such as gonadoblastoma and dysgerminoma. Gonadoblastoma is a benign gonadal tumor with a high potential for malignancy; it can differentiate into invasive dysgerminoma in 60% of cases and also in other forms of malignant tumors. About 90% of patients with gonadoblastoma have Y-chromosome material in their genetic makeup. Therefore, sequence detection of Y-chromosome by cytogenetic and/or molecular techniques in patients with TS is critical. In positive cases, prophylactic removal of gonads has been indicated.^4^ Two recently published retrospective studies showed Y chromosome material frequencies in TS by classical cytogenetics of 6.6% (4/61)^5^ and 7.6% (12/158).^6^ In one such study, 33% of patients (4/12) had gonadoblastoma and in two patients it progressed to disgerminona or teratoma.^6^ In another study with 11 patients with sexual differentiation disorders, 7 had Turner phenotype and mosaic karyotype Y in peripheral blood.^7^ All patients with TS underwent gonadectomy, and histopathological findings revealed that four of them (57.1%) had gonadoblastoma, and in two cases it was associated with dysgerminoma.^7^ Regarding the classical cytogenetic analysis by GTG banding, peripheral blood lymphocytes are the material of choice because it is easy to harvest this tissue, and the analysis is usually performed in 30 metaphases, which allows detection of 10% of mosaicism.^8^ The advantage of molecular methods is that it require no cell culture and only a small sample for analysis and are more sensitive to detect low mosacismo, frequent in TS.^8^

Thus, the aim of this review is to present the prevalence of Y chromosome sequences by molecular techniques and gonadoblastoma in patients with TS.

## Method

**Figure 1:**
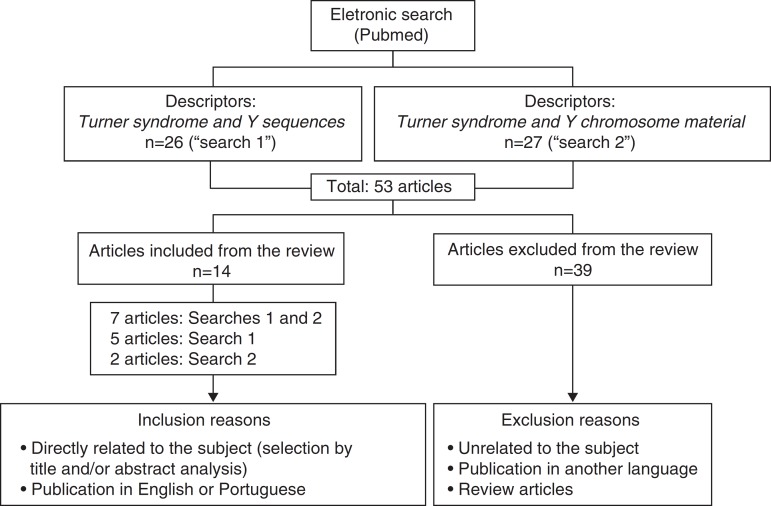
Representation of the method and electronic search results.

A literature search was performed on Pubmed, on 10/24/2014, with time limit between 2005 and 2014. Fig. 1 shows the flowchart of this electronic search.

## Results

Table 1 shows the frequency of Y chromosome sequences, identified by molecular techniques, of the 14 selected studies. Table 2 shows the karyotype of these patients. The frequency of gonadoblastoma in TS patients with positive amplification for Y-chromosome is summarized in Table 3, with information on gonadectomy and gonadoblastoma in TS in 9 of 14 studies. The results of five studies^3^^,^ 14 ^,^ 15 ^,^ 17 ^,^ 21 are not included in Table 3 for the following reasons: (1) two studies had no information on gonadectomy and gonadoblastoma in the text^3^^,^ 21; (2) in two studies patients were scheduled for clinical follow-up^17^ or monitoring by a multidisciplinary team of biologist, psychologist, geneticist, endocrinologist, and gynecologist^14^; and (3) in one study prophylactic gonadectomy was offered to all patients with Y sequences, however, the same patients opted for regular monitoring by ultrasound and CT.^15^

**Table 1:** Frequency of Y-chromosome sequences identified by molecular techniques in patients with Turner syndrome.

Reference	Method(s) and Y-chromosome sequence(s) and/or probe used	n/origin	PY	Frequency
Bianco et al. 9	PCR: *SRY/DYZ3*	20/Brazil	7	35.0%
Bianco et al. 10	PCR: *SRY/DYZ3*	5/Brazil	3	60.0%
Bianco et al. 11	PCR: *SRY/DYZ3/TSPY*	87/Brazil	16	18.5%
Bianco et al. 12	PCR: *SRY/TSPY*	104/Brazil	17	16.3%
Barros et al. 3	PCR and PCR nested: *SRY/TSPY/DYZ3*	96/Brazil	10	10.4%
Barros et al. 13	PCR and PCR nested: *SRY/TSPY/DYZ3* ; FISH: *DXZ1/DYZ3*	101/Brazil	16	15.8%
Araujo et al. 14	PCR: *SRY/ZFY/DYZ1/PABY/DYS1/DYZ3*	42/Brazil	2	4.8%
Bispo et al. 15	FISH: CEP X/CEP Y; PCR multiplex: *SRY/TSPY/AMGY/DAZ*	74/Brazil	5	6.8%
Mazzanti et al. 16	PCR: *SRY/DYZ3* ; FISH: CEP 18 SA/X SG/Y SO	171/Itly	14	8.2%
Semerci et al. 17	PCR: *PABY/SRY/DYS14/AMGY/DYZ3/DYS273/DYS280/DYS218/DYS224/DYS209/DYS231/DYS1/YRRM/DYZ1*	40/Turkey	2	5.0%
Sallai et al. 18	RT-PCR: *SRY/DDX3Y/HSFY1/TSPY* ; FISH: *whole chromosome painting Y/wcp*	130/Hungary	9 (RT-PCR)	6.9%
			6 (FISH)	4.6%
Cortés-Gutiérrez et al. 19	PCR: *SRY* ; FISH: CEP-Y	32/Mexico	3	9.4%
Freriks et al. 20	FISH: CEP X/CEP Y/ *SRY* ; PCR: Yp ( *SRY* ) and Yq ( *sY84/sY86/sY127/sY134/sY254/sY255* ); RT-PCR: *SRY/DSY14*	63/Holland	5	7.9%
Knauer-Fischer et al. 21	FISH: Ycen-1089/1090; PCR Multiplex: Yp11 (GBY: *TSPY/SRY* ), Yq11 (AZFa/AZFb/AZFc)	60/Germany	7	11.7%

PY, patients with Y sequence; PCR, polymerase chain reaction; SRY, sex-determining region on the Y chromosome or sex related region Y; FISH, fluorescence in situ hybridization; RT-PCR real-time PCR; DDX3Y, DEAD/H box polypeptide, Y-chromosome; HSFY1, heat-shock transcription factor, Y-linked; AZF, azoospermia factor; CEP X, X-specific centromeric probe; CEP Y, Y-specific centromeric probe; GBY, gonadoblastoma Y gene; TSPY, testis-specific protein Y-encoded or testis specific protein Y.

**Table 2:** Karyotypes determined by conventional cytogenetics (GTG) in patients with Y-chromosome sequences identified by molecular techniques.

Reference	Karyotypes
Bianco et al. 9	45,X: n=7
Bianco et al. 10	45,X/46,X,+mar: n=2; 45,X/45,X,add(15)(p11): n=1
Bianco et al. 11	45,X: n=12; 45,X/46,X,+mar: n=2; 45,X/45,X,add(15)(p11): n=1; 45,X/46,X,r(?): n=1
Bianco et al. 12	45,X: n=12; 45,X/46,X,+mar: n=2; 45,X/45,X,add(15)(p11): n=1; 45,X/46,X,r(?): n=1; 45,X/47,XXX: n=1
Barros et al. 3	45,X/46,X,+mar: n=5; 45,X: n=3; 45,X/46,X,r(?): n=2
Barros et al. 13	45,X: n=3; 45,X/46,XY: n=5; 45,X/46,X,+mar: n=5; 45,X/46,X,r(?): n=2; 45,X/47,XYY: n=1
Araujo et al. 14	45,X: n=2
Bispo et al. 15	45,X/46,XY: n=2; 45,X: n=1; 45,X/46,XY: n=1; 46,X,i(Xq): n=1
Mazzanti et al. 16	45,X/46,XY: n=6; 45,X: n=2; 45,X/46,X,idic(Y): n=3; 45,X/46,X,+mar: n=2; 45,X/46,XY/46,X,idic(Y): n=1
Semerci et al. 17	45,X: n=2
Sallai et al. 18	45,X: n=3; 45,X/46,XY: n=3; 45,X/46,X,+mar: n=2; 45,X/46,X,del(Xq): n=1
Cortés-Gutiérrez et al. 19	45,X/46,X,+mar: n=1; 45,X/46,XY: n=1; 45,X: n=1
Freriks et al. 20	45,X: n=5
Knauer-Fischer et al. 21	45,X: n=2; 45,X/46,X,idicY(q11.2): n=2; 46,X,i(X)(q10): n=1; 46,X,del(X)(q12 ou q13.1): n=1; 46,X,der(X)t(X;Y)(p22.3;q11.21): n=1

**Table 3:** Frequency of gonadoblastoma in patients with Turner syndrome and Y-chromosome sequences.

Reference	PY/P total	PG/PY	PG operated	% With gonadoblastoma	PG age (years)
Bianco et al. 9	7/20	4/7 (57.1)	1	25.0	16
Bianco et al. 10	3/5	3/3 (100)	0	0	–
Bianco et al. 11	16/87	11/16 (68.7)	2	18.2	16 and 19 a
Bianco et al. 12	17/104	12/17 (70.6)	2	16.7	16 and 19 a
Barros et al. 13	16/101	16/16 (100)	3	18.8	15.9 b ( *OCT4* +: GL); 18.2 b ( *OCT4* +: GR); 17.7 b ( *OCT4* +: GR and GL)
Mazzanti et al. 16	14/171	12/14 (85.7)	4	33.3	7.64 and 2.8: GBB b 15.9 and 11.6: GBM b
Sallai et al. 18	9/130	9/9 (100)	1	11.1	5.5 b
Cortés-Gutiérrez et al. 19	3/32	3/3 (100)	1	33.3	10
Freriks et al. 20	5/63	4/5 (80)	0	0	–

a Age at diagnosis.

b Age at gonadectomy.

PY/P total, patients with Y sequence in relation to the total number of patients; PG/PY, gonadoblastoma patients compared to patients with Y sequence; PG operated, gonadoblastoma patients undergoing surgery; *OCT4* , octamer-binding transcription factor 4; G, gonad; R, right; E, left; GBB, bilateral gonadoblastoma; GBM, gonadoblastoma monolateral.

## Discussion

The present literature review study showed the prevalence of Y-chromosome sequences and the risk of developing gonadoblastoma in TS patients.

### Y-chromosome sequences

Of the 14 studies included in this review, 60% were performed by Brazilian investigators.^3^^,^ 9 ^–^ 15 Four have been published by the same group and the two studies reporting a higher prevalence of Y-chromosome sequences^9^^–^ 12 analyzed samples from different tissues (peripheral blood, oral mucosa cells, and hair root).^9^^,^ 10 One study evaluated 20 patients with TS and karyotype 45,X^9^ and the other 5 patients with chromosomal abnormalities, such as chromosome marker, additional material, or ring chromosome.^10^ Three studies^9^^–^ 11 showed that all investigated patients had amplification for *SRY* gene and only a few for *DYZ3* and *TSPY*. This data shows that the *SRY* gene should be especially investigated in patients with TS.^9^^–^ 11 In a study published in 2010, Bianco et al.^12^ also evaluated gene expression (*SRY, TSPY, SF1, WT1, DAX1, OCT4, GATA4, FOG2, STRA8*) in right and left gonads of six patients undergoing gonadectomy and found no difference in the expression of these genes in this tissue both in patients and controls, except in one case with high expression of genes SRY, TSPY, and OCT4 in both gonads [karyotype:45,X/45,X,add(15)(p11)]. These nine investigated genes are involved in sex determination, differentiation, and gonadal tumorigenesis.^12^ In this study,^12^ all patients showed *SRY* gene amplification, which highlight the findings previously publicados.^9^^–^ 11 The studies presented above used exclusively the polymerase chain reaction (PCR) technique for the investigation of Y-chromosome sequences in TS^9^^–^ 12 and reported frequencies ranging from 16.3 to 60%.

Two studies performed by another group of investigators also used PCR and PCR in it.^3^^,^ 13 One of them assessed Y-chromosome sequences in 96 TS patients a total of 260.^3^ This research was indicated only in cases of: (a) 45,X karyotype and negative X-chromatin; (b) presence of marker chromosomes in karyotype, i.e., chromosome fragments of unknown origin; and (c) impossibility of cytogenetic identification of ring chromosome origin. Thus, 96 patients met these criteria and of these 10 showed positive amplification for the investigated genes. In this same study, six cases of intact Y-chromosome had previously been identified by classical cytogenetics (45,X/46,XY: n=5 and 45,X/47,XYY: n=1). In a subsequent study, these same authors evaluated 101 of 260 TS patients by PCR and, when positive, they used the fluorescence in situ hybridization (FISH) technique.^13^ The 101 cases analyzed by PCR for Y-chromosome sequences showed 45, X karyotype and negative Barr corpuscle (n=73), marker chromosome (n=25), and ring chromosome (n=3).

Two other studies reported the lower Y-chromosome frequency values among studies performed in Brazil.^14^^,^ 15 In four TS patients, the detection of Y-chromosome sequences was identified only by PCR [karyotypes: 45,X and 46,X,i(Xq)].^14^^,^ 15 Thus, the inclusion of PCR technique in TS routine investigation would be indicated.^14^ It is noteworthy that six Brazilian studies have used only the PCR technique for the investigation of Y-chromosome sequences. Advantages of this technique include: (1) quick execution; (2) low cost; (3) simultaneous processing of multiple samples; (4) applicability in screening of a large number of patients; and (5) high sensitivity for detection of mosaicism. The use of PCR to investigate 14 Y-chromosome sequences identified a prevalence of 5%,^17^ value similar to that reported by a study analyzing only six sequences.^14^

FISH and PCR techniques were concurrently used in several studies.^15^^,^ 16 ^,^ 18 ^–^ 21 One study published in 2005 identified a frequency of about 6% by classical cytogenetics (10/171) and 8% by molecular analysis (14/171).^16^ In another study,^19^ GTG banding revealed three positive cases: karyotypes 45,X/46,X,mar; 45,X/46,XY, and 45,X. FISH analysis of lymphocytes revealed: (1) that the marker chromosome was Y; (2) confirmed the Y-chromosome previously identified by classical cytogenetics; and (3) did not identify Y-chromosome material, respectively. FISH analysis of gonadal tissue showed that Y-chromosome was present in 57, 46 and 26% of cells, respectively. Investigation by PCR/SRY in lymphocytes and gonads was positive in all three cases. The case considered Y-negative in lymphocytes by GTG and FISH (45,X) was positive for SRY by PCR/lymphocytes. Therefore, the authors suggested: (1) the use of PCR, in addition to conventional cytogenetic analysis, to rule out the possibility of hidden Y-chromosome mosaicism; and (2) the use of FISH technique only after a positive result for PCR because it is expensive and laborious.^19^ In addition to FISH, real-time PCR (RT-PCR) was also used.^18^^,^ 20 The first study^18^ identified Y-chromosome mosaicism by conventional cytogenetic analysis in three of the 130 patients investigated (karyotypes: 45,X/46,XY n=3). Two patients have mosaicism around the Y-chromosome, while the other patient had only part of the Y-chromosome. By RT-PCR, nine patients were positive for Y. Of the four Y-chromosome specific probes used in the RT-PCR technique, two (*SRY* and *TSPY1*) are on the Y-chromosome short arm (Yp) and two (*DDX3Y* and *HSFY1*) on the long arm (Yq). Six patients had amplification to the four sequences, three to two (*SRY* and *TSPY1*) or three (*SRY, TSPY1*, and *DDX3Y*) sequences; that is, three patients had no complications of *HSFY1* gene and one had loss of *DDX3Y* region, which suggests Yq deletion. FISH analysis confirmed positive for Y in six cases. Thus, the frequency of Y-chromosome sequences was 2.3, 4.6, and 6.9% by GTG bands, RT-PCR, and FISH techniques, respectively. In the 2013 study,^20^ with 162 TS patients, 75 had a 45,X karyotype and of these 63 have agreed to undergo additional molecular investigations. Y sequences were identified by FISH in five patients on oral cell samples but not in peripheral blood lymphocytes by PCR. RT-PCR analysis revealed the presence of *SRY* and *DYS14* in two of them.^20^ In another study, the combined use of PCR and FISH allowed the identification of a frequency of approximately 12% of Y-chromosome sequences in TS. Of the seven patients with Y-sequences,^21^ four did not show it in the karyotype.

In short, in the present study the frequency of Y-chromosome sequences in TS patients ranged from 4.6 to 60%. According to the literature, the prevalence is 0–61%.^19^ This difference may be due to the following factors: selection criteria of patients, sample size, methodology, and Y-chromosome markers used. When the analyzed tissue was peripheral blood alone, the prevalence of Y sequences ranged from 4.6 to 18.5%.^11^^,^ 21 However, in studies where more than one tissue evaluated, the prevalence was greater.^9^^,^ 10 *SRY, DYZ3*, and *TSPY* genes were the most widely investigated. *SRY* gene is located in the short arm of Y-chromosome (Yp11.3) and *DYZ3* gene in the pericentromeric region at Yp12. Both genes play a role in sex determination and chromosomal stability, respectively.^22^ *TSPY* (testis-specific protein Y encoded), located in the gonadoblastoma region of the Y-chromosome (GBY) at Yp11.2, is involved in the development of gonadoblastoma and its expression was detected in this tissue and testis.^23^^–^ 25 Regarding technique, seven studies^3^^,^ 9 ^–^ 14 ^,^ 17 used PCR alone to investigate Y-chromosome sequences in TS and in the remaining seven studies FISH was also used.^13^^,^ 15 ^,^ 16 ^,^ 18 ^–^ 21 An intriguing finding was reported by one of these studies, which found Y-negative sequences in all 56 cases, including the five positive cases by FISH/buccal cells.^20^ This result contradicts those published in the literature that recommend the use of PCR in the investigation of Y-chromosome sequences in TS. The authors attribute this low sensitivity to the difficulty in detecting mosaicism below 10%.^20^ Chromosomal mosaicism is defined as the presence of two or more distinct cell lines within the same individual resulting from post-zygotic nondisjunction. When it occurs in already differentiated cells, mosaicism may be confined to one or a few tissues.^26^ In this context, investigation of Y-chromosome sequences in tissues of different embryonic origins is advised, such as cells of the oral mucosa, which can easily be harvested by non-invasive procedures.

To our knowledge, there is only one systematic review focusing on clinical and genetic characteristics of TS, mosaicism, Y-chromosome, and risk of gonadal tumor.^8^ Data presented in this study show: (1) that the detection of Y-chromosome sequences in TS, regardless of the karyotype, is necessary to prevent the development of gonadoblastoma; and (2) PCR technique should be employed due to its high sensitivity, low cost, and easy to perform.

After TS confirmation by cytogenetics, the inclusion of the PCR technique is suggested as a complement for detection of Y-chromosome sequences in these patients. This molecular technique is more sensitive and can detect the presence of Y-chromosome material.

### Gonadoblastoma

Gonadoblastoma, often observed in the second decade of life, is a benign gonadal tumor with a high potential for malignant transformation.^4^ It has a good prognosis and may differentiate into a germ cell tumor, such as dysgerminomas, and less frequently into teratomas, embryonal carcinoma, yolk sac tumor, and choriocarcinoma.^13^ This tumor is present mainly in women with gonadal dysgenesis, and approximately 95% of them have Y-chromosome material in their genomes. Therefore, the detection of these sequences by cytogenetic and/or molecular techniques has been encouraged to guide the prophylactic indication for surgical removal of gonads in this group of patients, as generally they are not metastatic tumors, and there is the possibility of cure with their removal.^4^ Women with TS have gonadal dysgenesis and therefore the detection of the Y-chromosome sequences in these patients is of extreme clinical importance.

The highest prevalence for gonadoblastoma in this review was 33%^16^^,^ 19 and is in line with other studies.^6^^,^ 27 This percentage was identified in a recent survey, and four patients with ST had the karyotype 45,X/46,XY.^6^ Two of them had a dysgerminoma (age at surgery: 11.25 years) and a teratoma (15 years). In the two patients with gonadoblastoma alone, age at surgery was 1.5 and 11.7 years.^6^ Another study published recently reported 35.3% incidence of gonadoblastoma.^28^ Twenty patients with TS had Y-chromosome sequences from a total of 217 and 17 of them underwent gonadectomy.^28^ An even higher frequency was reported by Alvarez-Nava et al.^29^ who investigated 52 patients and detected four with Y-chromosome sequences (7.7%), all underwent gonadectomy and two had gonadoblastoma (50%). Five studies reported incidence of 10–25%.^9^^,^ 11 ^–^ 13 ^,^ 18 Previous studies have reported frequencies of 7–10%^30^ and 16.7%.^31^ These findings show that the frequency of gonadoblastoma is variable and may reflect an early investigation.

In studies performed by Bianco et al.,^9^^–^ 12 results obtained were: (1) one 45,X patient with bilateral gonadoblastoma positive *SRY* gene amplification for the three tissues analyzed^9^; (2) two patients with bilateral gonadoblastoma [45,X: SRY+ and 45,X/46,X,r(?): *SRY*+*DYZ3*+]^11^; (3) two patients with bilateral gonadoblastoma [45,X: *SRY*+ and 45,X/46,X,r(?): *SRY*+].^12^ Although there was no patient identified with gonadoblastoma, histopathological study of the gonads of one patient revealed stromal cell hyperplasia and luteoma.^10^ Subsequent studies also showed patients with these histopathological findings.^11^^,^ 12

Another gene associated with gonadoblastoma is *OCT4* (octamer-binding transcription factor 4),^12^^,^ 13 also known as *OCT3* or *POU5FI*. This gene is considered to be a tumor marker of germ cells, such as gonadoblastoma, dysgerminoma, seminoma, and others, and its expression was detected by immunohistochemistry in 100% of cases of gonadoblastoma.^32^ The study published in 2010 showed high expression of the *SRY, TSPY*, and *OCT4* genes in both gonads of a patient with positive PCR (*SRY*+ and *TSPY*+).^12^ Another study showed that immunohistochemical analysis for *OCT4* was positive in three cases (karyotypes: 45, X/46,XY: n=2 and 45,X/46,X,+mar: n=1), a suggesting result of germ cell tumor (gonadoblastoma or carcinoma in situ).^13^ In this study, the 16 cases of Y sequences underwent bilateral gonadectomy, and the left and right gonads were assessed by conventional staining with hematoxylin and eosin (H&E) and immunohistochemical staining for *OCT4*. Gonadal neoplasia was not detected in any of 32 gonads evaluated by H&E; however, four gonads (12%) of three patients (19%) were positive for *OCT4*, suggesting the existence of germ cell tumors.^13^ For this reason, the authors recommend a specific histopathological study in gonads, such as *OCT4* immunohistochemistry, to assess the real risk of gonadal tumors in patients with TS and Y-chromosome sequences.^12^^,^ 13

Regarding the studies that reported a higher prevalence of gonadoblastoma, one of them reported two patients with monolateral gonadoblastoma (karyotype: 45,X and 45,X/46,X,+mar) and two with bilateral gonadoblastoma (karyotype: 45,X/46,XY)^16^ and the other reported a patient 45,X/46,XY with gonadoblastoma.^19^ In the study published in 2010, prophylactic surgery was performed in all nine patients with Y-chromosome material before age of 20 and one of them, a girl of 5-year old, the youngest patient in the study, had bilateral gonadoblastoma without clinical signs.^18^ This patient had a 45,X/46,XY karyotype (GTG), wcpY+^93^ (FISH), and was positive for all Y-specific sequences (*SRY, TSPY1, DDX3Y*, and *HSFY1*). Ovarian tissue histology revealed no gonadoblastoma in four of the patients undergoing gonadectomy.^20^

A recent histopathological study reported gonadal tumors in 6 out of 11 patients (56%), including 4 out of 7 (57%) with TS.^7^ Two patients with TS had gonadoblastoma in the right and left gonads [karyotypes and age at gonadectomy, respectively: 45,X/45,X,t (15; Y)(p11.2; q11.2) and 2 years and 11 months; 45,X/46,XY and 10 years and 3 months]. Gonadoblastoma associated with dysgerminoma was seen in only one gonad (right, in a patient; and left, in other) and in two other patients (karyotype 45,X/46,XY and 46,X,+der(15)/46,XY and ages: 11 years and 6 months and 15 years and 4 months, respectively). The youngest patient with TS submitted to gonadectomy was 2 years old.^7^

According to data presented, it can be concluded that molecular investigation is indicated for Y-chromosome sequences in TS patients, regardless of the karyotype, as a complement to the cytogenetic diagnosis. PCR is the technique suggested because it is inexpensive, sensitive, rapid, and enable the tracking of various sequences of Y-chromosome simultaneously. It would also be appropriate for analysis of a second tissue, in addition to peripheral blood. In those patients with Y-positive sequences, gonadoblastoma investigation is required.
