# Supplementary material for: Prevalence of Y-chromosome sequences and gonadoblastoma in Turner syndrome
Source: Rev Paul Pediatr. 2016 Jan-Mar;34(1):114–21. doi: 10.1016/j.rppede.2015.12.004 (PMC4795730; doi:10.1016/j.rppede.2015.12.004)
Supplement: Translation

# Translation: Prevalência de sequências do Y e de gonadoblastoma em síndrome de Turner

**Authors:** Alessandra Bernadete Trovó de Marqui, Roseane Lopes da Silva-Grecco, Marly Aparecida Spadotto Balarin

**Affiliations:** Universidade Federal do Triângulo Mineiro (UFTM), Uberaba, MG, Brasil

**Keywords:** Síndrome de Turner, Cromossomo Y, Gonadoblastoma, Prevalência, Reação em cadeia da polimerase, Disgenesia gonadal

## Abstract

**Objetivo:**

Apresentar a prevalência de sequências do cromossomo Y por técnicas moleculares e de gonadoblastoma em pacientes com síndrome de Turner.

**Fontes de dados:**

Foi feita uma pesquisa bibliográfica no Pubmed, com limite de período entre 2005 e 2014, com os descritores *Turner syndrome* and *Y sequences* (n=26) e *Turner syndrome* and *Y chromosome material* (n=27). Os critérios de inclusão foram artigos que tivessem relação direta com o tema e publicados no idioma inglês ou português. Foram excluídos aqueles que não cumpriram esses critérios e eram do tipo revisão. Após aplicação desses critérios, 14 foram selecionados.

**Síntese dos dados:**

Os principais resultados quanto à prevalência de sequências do cromossomo Y em síndrome de Turner foram: 1 – cerca de 60% dos estudos foram feitos por pesquisadores brasileiros; 2 – a frequência variou de 4,6 a 60%; 3 – os genes *SRY, DYZ3* e *TSPY* foram os mais investigados; 4 – a técnica de PCR foi empregada exclusivamente em sete estudos e nos sete restantes, associada à FISH. Nove dos 14 estudos apresentaram informações sobre gonadectomia e gonadoblastoma. Dois estudos relataram a maior prevalência para gonadoblastoma (33%). Cinco dos nove estudos referiram prevalência de 10 a 25% e em dois esse valor foi nulo.

**Conclusões:**

De acordo com os dados apresentados, é indicada a pesquisa molecular para sequências do cromossomo Y em pacientes com ST, independentemente do cariótipo. Naquelas com positividade para essas sequências, é necessária a investigação de gonadoblastoma.

## Introdução

A síndrome de Turner (ST) é um distúrbio cromossômico com incidência de uma a cada 2.500 meninas; sua etiologia está associada à monossomia total ou parcial do cromossomo X e o diagnóstico é feito pelo exame do cariótipo.^1^^,^ 2 Um estudo retrospectivo em 260 pacientes com ST mostrou que o aprimoramento da análise cromossômica promoveu uma modificação da proporção dos tipos de cariótipos observados.^3^

As pacientes com ST exibem como principais sinais clínicos baixa estatura e disgenesia gonadal. Também podem apresentar implantação baixa de cabelos na nuca, estrabismo, ptose palpebral, palato ogival, micrognatia, pescoço curto e/ou alado, linfedema de mãos e/ou pés, encurtamento de metacarpianos e/ou metatarsianos, deformidade de Madelung, *cubitus valgus, genu valgum*, escoliose e múltiplos nevos pigmentados, anomalias cardiovasculares e renais, doenças tireoidianas, deficiência auditiva, hipertensão, osteoporose e obesidade.^1^^,^ 2 No entanto, essa síndrome é caracterizada por ampla variabilidade fenotípica, desde pacientes com fenótipo clássico até aquelas quase indistinguíveis da população geral.

Mulheres com ST que apresentam material do cromossomo Y têm risco aumentado de desenvolver tumores gonadais, tais como gonadoblastoma e disgerminoma. O gonadoblastoma é um tumor gonadal benigno com um alto potencial de transformação maligna, pode diferenciar-se em disgerminoma invasivo em 60% dos casos e, também, em outras formas malignas de tumores. Cerca de 90% dos pacientes com gonadoblastoma têm material do cromossomo Y em sua constituição genética. Por esse motivo, a detecção de sequências do cromossomo Y, por técnicas citogenéticas e/ou moleculares, em pacientes com ST é de extrema importância clínica. Em casos positivos, tem sido indicada a cirurgia profilática para retirada das gônadas.^4^ Dois estudos retrospectivos publicados recentemente revelaram frequências de material do cromossomo Y em ST por citogenética clássica de 6,6% (4/61)^5^ e 7,6% (12/158).^6^ Em um desses estudos, 4/12 (33%) das pacientes exibiram gonadoblastoma e em duas esse evoluiu para disgerminona ou teratoma.^6^ Em outro, com 11 pacientes com distúrbios de diferenciação sexual, sete mostraram fenótipo Turner e cariótipo mosaico de Y em sangue periférico.^7^ Todas as pacientes com ST foram submetidas à gonadectomia e os achados histopatológicos revelaram que quatro delas (57,1%) tinham gonadoblastoma e em dois casos associação desse com disgerminoma.^7^ Ainda em relação à citogenética clássica por bandamento GTG, linfócitos de sangue periférico constituem o material de escolha devido à facilidade de obter esse tecido e a análise é, geralmente, feita em 30 metáfases, o que permite a detecção de 10% de mosaicismo.^8^ Os métodos moleculares têm como vantagem a não necessidade de cultura celular e de apenas uma pequena quantidade de amostra para análise e são mais sensíveis para detecção de baixo mosacismo, frequente em ST.^8^

Desse modo, o objetivo desta revisão é apresentar a prevalência de sequências do cromossomo Y por técnicas moleculares e gonadoblastoma em pacientes com ST.

## Método

Foi feita uma pesquisa bibliográfica no Pubmed, em 24/10/2014, com limite de período entre 2005 e 2014. A figura 1 apresenta o fluxograma dessa busca eletrônica.

**Figura 1:** Representação do método e resultados da busca eletrônica.

## Resultados

A tabela 1 apresenta a frequência de sequências do cromossomo Y, identificada por técnicas moleculares, dos 14 estudos selecionados. O cariótipo dessas pacientes encontra-se na tabela 2. A frequência de gonadoblastoma, em pacientes com ST com amplificação positiva para o cromossomo Y, consta sumarizada na tabela 3. Essa apresenta informações sobre gonadectomia e gonadoblastoma em ST em nove dos 14 estudos. Os resultados de cinco estudos não constam na tabela 3 3 ^,^ 14 ^,^ 15 ^,^ 17 ^,^ 21  pelos seguintes motivos: 1 –dois estudos não apresentaram informações sobre gonadectomia e gonadoblastoma no texto;^3^^,^ 21 2 – em dois, os pacientes foram agendados para seguimento clínico^17^ ou acompanhamento por equipe multidisciplinar composta por biólogo, psicólogo, geneticista, endocrinologista e ginecologista^14^ e 3 – no outro estudo, a gonadectomia profilática foi oferecida a todas as pacientes que apresentaram sequências do Y, entretanto, as mesmas optaram por acompanhamento periódico por ultrassonografia e tomografia.^15^

**Tabela 1:** Frequência de sequências do cromossomo Y, identificada por técnicas moleculares, em pacientes com síndrome de Turner

Referência	Método e sequência(s) do cromossomo Y e/ou sonda(s) utilizada(s)	n/Origem	PY	Frequência
Bianco *et al* . 9	PCR: *SRY/DYZ3*	20/Brasil	7	35,0%
Bianco *et al* . 10	PCR: *SRY/DYZ3*	5/Brasil	3	60,0%
Bianco *et al* . 11	PCR: *SRY/DYZ3/TSPY*	87/Brasil	16	18,5%
Bianco *et al* . 12	PCR: *SRY/TSPY*	104/Brasil	17	16,3%
Barros *et al* . 3	PCR e PCR nested: *SRY/TSPY/DYZ3*	96/Brasil	10	10,4%
Barros *et al* . 13	PCR e PCR nested: *SRY/TSPY/DYZ3* ; FISH: *DXZ1/DYZ3*	101/Brasil	16	15,8%
Araújo *et al* . 14	PCR: *SRY/ZFY/DYZ1/PABY/DYS1/DYZ3*	42/Brasil	2	4,8%
Bispo *et al* . 15	FISH: CEP X/CEP Y; PCR multiplex: *SRY/TSPY/AMGY/DAZ*	74/Brasil	5	6,8%
Mazzanti *et al* . 16	PCR: *SRY/DYZ3;* FISH: CEP 18 SA/X SG/Y SO	171/Itália	14	8,2%
Semerci *et al* . 17	PCR: *PABY/SRY/DYS14/AMGY/DYZ3/DYS273/DYS280/DYS218/DYS224/DYS209/DYS231/DYS1/YRRM/DYZ1*	40/Turquia	2	5,0%
Sallai *et al* . 18	RT‐PCR: *SRY/DDX3Y/HSFY1/TSPY;* FISH: *whole chromosome painting Y/wcp*	130/Hungria	9 (RT‐PCR)	6,9%
			6 (FISH)	4,6%
Cortés‐Gutiérrez *et al* . 19	PCR: *SRY;* FISH: CEP‐Y	32/México	3	9,4%
Freriks *et al* . 20	FISH: CEP X/CEP Y/ *SRY* ; PCR: Yp ( *SRY* ) e Yq ( *sY84/sY86/sY127/sY134/sY254/sY255* ); RT‐PCR: *SRY/DSY14*	63/Holanda	5	7,9%
Knauer‐Fischer *et al* . 21	FISH: Ycen‐1089/1090; PCR Multiplex: Yp11 (GBY: *TSPY/SRY* ), Yq11 (AZFa/AZFb/AZFc)	60/Alemanha	7	11,7%

PY, pacientes com sequência Y; PCR, Polimerase Chain Reaction – Reação em Cadeia da Polimerase; SRY, sex‐determining region on the Y chromosome ou sex releated region Y; Fish, Fluorescence in situ hybridization – Hibridização *in situ* fluorescente; RT‐PCR, Real‐time PCR; DDX3Y, DEAD/H box polypetide, Y‐chromosome; HSFY1, heat‐shock transcription factor, Y‐linked; AZF, azoospermia factor; CEP X, X‐specific centromeric probe; CEP Y, Y‐specific centromeric probe; GBY, gonadoblastoma Y gene; TSPY, testis‐specific protein Y‐encoded ou testis specific protein Y.

**Tabela 2:** Cariótipos determinados por citogenética convencional (GTG) nas pacientes com sequências do cromossomo Y identificadas por técnicas moleculares

Referência	Cariótipos
Bianco *et al* . 9	45,X: n=7
Bianco *et al* . 10	45,X/46,X,+mar: n=2; 45,X/45,X,add(15)(p11): n=1
Bianco *et al* . 11	45,X: n=12; 45,X/46,X,+mar: n=2; 45,X/45,X,add(15)(p11): n=1; 45,X/46,X,r(?): n=1
Bianco *et al* . 12	45,X: n=12; 45,X/46,X,+mar: n=2; 45,X/45,X,add(15)(p11): n=1; 45,X/46,X,r(?): n=1; 45,X/47,XXX: n=1
Barros *et al* . 3	45,X/46,X,+mar: n=5; 45,X: n=3; 45,X/46,X,r(?): n=2
Barros *et al.* 13	45,X: n=3; 45,X/46,XY: n=5; 45,X/46,X,+mar: n=5; 45,X/46,X,r(?): n=2; 45,X/47,XYY: n=1
Araújo *et al.* 14	45,X: n=2
Bispo *et al* . 15	45,X/46,XY: n=2; 45,X: n=1; 45,X/46,XY: n=1; 46,X,i(Xq): n=1
Mazzanti *et al* . 16	45,X/46,XY: n=6; 45,X: n=2; 45,X/46,X,idic(Y): n=3; 45,X/46,X,+mar: n=2; 45,X/46,XY/46,X,idic(Y): n=1
Semerci *et al.* 17	45,X: n=2
Sallai *et al* . 18	45,X: n=3; 45,X/46,XY: n=3; 45,X/46,X,+mar: n=2; 45,X/46,X,del(Xq): n=1
Cortés‐Gutiérrez *et al.* 19	45,X/46,X,+mar: n=1; 45,X/46,XY: n=1; 45,X: n=1
Freriks *et al* . 20	45,X: n=5
Knauer‐Fischer *et al* . 21	45,X: n=2; 45,X/46,X,idicY(q11.2): n=2; 46,X,i(X)(q10): n=1; 46,X,del(X)(q12 ou q13.1): n=1; 46,X,der(X)t(X;Y)(p22.3;q11.21): n=1

**Tabela 3:** Frequência de gonadoblastoma em pacientes com síndrome de Turner e sequências do cromossomo Y

Referência	PY/P total	PG/PY	PG operadas	% com gonadoblastoma	Idade da PG (anos)
Bianco *et al*. 9	7/20	4/7 (57,1)	1	25,0	16
Bianco *et al*. 10	3/5	3/3 (100)	0	0	–
Bianco *et al*. 11	16/87	11/16 (68,7)	2	18,2	16 e 19 a
Bianco *et al*. 12	17/104	12/17 (70,6)	2	16,7	16 e 19 a
Barros *et al*. 13	16/101	16/16 (100)	3	18,8	15,9 b (*OCT4* +: GE); 18,2 b ( *OCT4* +: GD); 17,7 b ( *OCT4* +: GD e GE)
Mazzanti *et al* . 16	14/171	12/14 (85,7)	4	33,3	7,64 e 2,8: GBB b 15,9 e 11,6: GBM b
Sallai *et al* . 18	9/130	9/9 (100)	1	11,1	5,5 b
Cortés‐Gutiérrez *et al* . 19	3/32	3/3 (100)	1	33,3	10
Freriks *et al* . 20	5/63	4/5 (80)	0	0	–

a Idade no diagnóstico.

b Idade na gonadectomia.

PY/P total, pacientes com sequência Y em relação ao total de pacientes; PG/PY, pacientes com gonadoblastoma em relação às pacientes com sequência Y; PG operadas, pacientes com gonadoblastomas submetidas à cirurgia; Pac, paciente; *OCT4 , octamer‐binding transcription factor 4* ; G, gônada; D, direita; E, esquerda; GBB, gonadoblastoma bilateral; GBM, gonadoblastoma monolateral.

## Discussão

O presente estudo, do tipo revisão bibliográfica, apresentou a prevalência de sequências do cromossomo Y e o risco de desenvolvimento de gonadoblastoma em pacientes com ST.

### Sequências do cromossomo Y

Dos 14 estudos inclusos nesta revisão, cerca de 60% foram feitos por pesquisadores brasileiros.^3^^,^ 9 ^-^ 15 Quatro foram publicados pelo mesmo grupo de pesquisadores^9^^-^ 12 e os dois que relataram maior prevalência de sequências do cromossomo Y analisaram amostras de diferentes tecidos (sangue periférico, células de mucosa oral e raiz de cabelo).^9^^,^ 10 Um deles avaliou 20 pacientes com ST e cariótipo 45,X^9^ e o outro, cinco com anomalias cromossômicas, tais como cromossomo marcador, material adicional ou cromossomo em anel.^10^ Três estudos^9^^-^ 11 mostraram que todas as pacientes investigadas apresentaram amplificação para o gene *SRY* e apenas algumas para *DYZ3* e *TSPY.* Esse dado mostra que especialmente o gene *SRY* deve ser investigado em pacientes com ST.^9^^-^ 11 Em seu estudo publicado em 2010, Bianco *et al*.^12^ também avaliaram a expressão gênica (*SRY, TSPY, SF1, WT1, DAX1, OCT4, GATA4, FOG2, STRA8*) nas gônadas (direita e esquerda) de seis pacientes submetidas à gonadectomia e não observaram diferença na expressão desses genes nesse tecido tanto em pacientes quanto em controles, exceto em um caso em que houve alta expressão dos genes *SRY, TSPY* e *OCT4* em ambas as gônadas [cariótipo: 45,X/45,X,add(15)(p11)]. Esses nove genes investigados estão envolvidos na determinação sexual, diferenciação e tumorigênese gonadal.^12^ Nesse estudo,^12^ todas as pacientes exibiram amplificação para o gene *SRY*, o que reforça os achados previamente publicados.^9^^-^ 11 Os estudos apresentados acima empregaram exclusivamente a técnica de reação em cadeia da polimerase (PCR) para investigação de sequências do cromossomo Y em ST^9^^-^ 12 e relataram frequências que variaram de 16,3 a 60%.

Dois estudos conduzidos por outro grupo de pesquisadores também usaram a PCR e PCR neste.^3^^,^ 13 Um deles analisou sequências do cromossomo Y em 96 pacientes com ST de um total de 260.^3^ Essa pesquisa foi indicada somente nos casos com: a) cariótipo 45,X e cromatina X negativa; b) presença no cariótipo de cromossomos marcadores, ou seja, fragmentos cromossômicos de origem indefinida e c) impossibilidade da identificação citogenética da origem de cromossomos em anel. Desse modo, 96 pacientes cumpriram esses critérios e dessas 10 exibiram amplificação positiva para os genes investigados. Nesse mesmo estudo, seis casos de cromossomo Y íntegro haviam sido identificados previamente por citogenética clássica (45,X/46,XY: n=5 e 45,X/47,XYY: n=1). Em um estudo posterior, esses mesmos autores avaliaram 101 pacientes de 260 com ST por PCR e quando positivo, empregaram a técnica de Fish (hibridização *in situ* fluorescente).^13^ Os 101 casos analisados por PCR para sequências do cromossomo Y apresentaram cariótipo 45,X e corpúsculo de Barr negativo (n=73), cromossomo marcador (n=25) e em anel (n=3).

Duas outras pesquisas referiram os menores valores de frequências do cromossomo Y dentre os estudos conduzidos no Brasil.^14^^,^ 15 Em quatro pacientes com ST, a detecção de sequências do cromossomo Y foi identificada apenas por PCR [cariótipos: 45,X e 46,X,i(Xq)].^14^^,^ 15 Assim, a inclusão da técnica de PCR na rotina de investigação na ST seria indicada.^14^ Vale destacar que seis estudos brasileiros empregaram exclusivamente a técnica de PCR para investigação de sequências do cromossomo Y. As vantagens dessa técnica incluem: 1 – rapidez de execução, 2 – baixo custo, 3 – processamento de diversas amostras simultaneamente, 4 – aplicabilidade em rastreamento (*screening*) de um grande número de pacientes e 5 – alta sensibilidade no reconhecimento do mosaicismo. O emprego da técnica de PCR para investigação de 14 sequências do cromossomo Y identificou uma prevalência de 5%,^17^ valor semelhante àquele referido por um estudo que analisou apenas seis sequências.^14^

As técnicas de Fish e PCR foram, concomitantemente, empregadas em vários estudos.^15^^,^ 16 ^,^ 18 ^-^ 21 Um deles, publicado em 2005, identificou uma frequência de aproximadamente 6% por citogenética clássica (10/171) e 8% por análise molecular (14/171).^16^ Em outro estudo^19^ o bandamento GTG revelou três casos positivos: cariótipos 45,X/46,X,mar; 45,X/46,XY e 45,X. A análise por Fish em linfócitos revelou: 1) que o cromossomo marcador era o Y, 2) confirmou o cromossomo Y identificado previamente por citogenética clássica e 3) não identificou material do cromossomo Y, respectivamente. A análise de Fish de tecido gonadal mostrou que o cromossomo Y estava presente em 57; 46 e 26% das células, respectivamente. A pesquisa por PCR/*SRY* em linfócitos e gônadas foi positiva nos três casos. O caso considerado Y negativo em linfócitos por GTG e Fish (45,X) apresentou positividade para o *SRY* por PCR/linfócitos. Diante disso, os autores sugeriram: 1 – o emprego da PCR, além da análise citogenética convencional, para descartar a possibilidade de mosaicismo oculto do cromossomo Y e 2 – o uso da técnica de Fish somente após um resultado positivo para PCR, pois ela é dispendiosa e laboriosa.^19^ Além de Fish, a técnica de PCR em tempo real (RT-PCR) também foi aplicada.^18^^,^ 20 O primeiro estudo^18^ identificou mosaicismo do cromossomo Y, por análise citogenética convencional, em três das 130 pacientes investigadas (cariótipos: 45,X/46,XY n=3). Duas pacientes exibiram mosaicismo de todo o cromossomo Y enquanto a outra, apenas de parte do Y. Por RT-PCR, nove pacientes apresentaram positividade para o Y. Das quatro sondas específicas do cromossomo Y usadas na técnica de RT-PCR, duas (*SRY* e *TSPY1*) estão no braço curto do cromossomo Y (Yp) e duas (*DDX3Y* e *HSFY1*) no braço longo (Yq). Seis pacientes tiveram amplificação para as quatro sequências, três para duas sequências (*SRY* e *TSPY1*) ou três (*SRY, TSPY1* e *DDX3Y*), ou seja, três pacientes não mostraram complicações do gene *HSFY1* e uma delas exibiu perda da região *DDX3Y*, o que sugere deleção de Yq. A análise por Fish confirmou positividade para o Y em seis casos. Desse modo, a frequência de sequências do cromossomo Y foi de 2,3%; 4,6% e 6,9% pelas técnicas de banda GTG, FISH e RT-PCR, respectivamente. No estudo de 2013^20^ com 162 pacientes com ST, 75 tinham cariótipo 45,X e dessas 63 concordaram em fazer investigações moleculares adicionais. Sequências do Y foram identificadas por Fish em cinco pacientes em amostras de células orais, mas não em linfócitos de sangue periférico por PCR. A análise por RT-PCR revelou a presença de *SRY* e *DYS14* em duas delas.^20^ Em outro estudo, o uso combinado de PCR e Fish permitiu a identificação de uma frequência de aproximadamente 12% de sequências do cromossomo Y em ST. Das sete pacientes com sequências do Y,^21^ quatro não o apresentavam no cariótipo.

Em suma, no presente estudo a frequência de sequências do cromossomo Y em pacientes com ST variou de 4,6% a 60%. Segundo a literatura, essa prevalência é de 0% a 61%.^19^ Essa diferença pode ser devida aos seguintes fatores: critérios de seleção das pacientes, tamanho da amostra, metodologia e marcadores do cromossomo Y usados. Quando o tecido analisado foi somente sangue periférico, a prevalência de sequências do Y variou de 4,6% a 18,5%.^11^^-^ 18 ^,^ 21 No entanto, nos estudos nos quais mais que um tecido foi avaliado, essa prevalência foi ainda maior.^9^^,^ 10 Os genes *SRY, DYZ3* e *TSPY* foram os mais amplamente investigados. O gene *SRY* está localizado no braço curto do cromossomo Y (Yp11.3) e o *DYZ3* na região pericentromérica em Yp12. Ambos os genes desempenham papel na determinação sexual e na estabilidade cromossômica, respectivamente.^22^ O *TSPY* (*testis-specific protein Y encoded*), localizado no loco do gonadoblastoma no cromossomo Y (GBY), em Yp11.2, está envolvido no desenvolvimento de gonadoblastoma e sua expressão foi detectada nesse tecido e em testículo.^23^^-^ 25 Quanto à técnica, sete estudos^3^^,^ 9 ^-^ 14 ^,^ 17 empregaram unicamente a PCR para investigação de sequências do cromossomo Y em ST e nos sete estudos restantes também Fish.^13^^,^ 15 ^,^ 16 ^,^ 18 ^-^ 21 Um achado intrigante foi relatado por um desses estudos que referiu negatividade para sequências do Y em todos os 56 casos, inclusive nos cinco positivos por FISH/células bucais.^20^ Esse resultado contradiz aqueles publicados na literatura que recomendam o uso da PCR na investigação de sequências do cromossomo Y em ST. Os autores atribuem essa sensibilidade baixa à dificuldade de detectar mosaicismo inferior a 10%.^20^ Mosaicismo cromossômico é definido como a presença de duas ou mais linhagens celulares diferentes em um mesmo indivíduo resultante de não disjunção pós-zigótica. Quando esse ocorre em células já diferenciadas, o mosaicismo pode ser confinado a um ou poucos tecidos.^26^ Nesse sentido, a investigação de sequências do cromossomo Y em tecidos com diferentes origens embrionárias é aconselhada, como, por exemplo, as células de mucosa oral, que podem ser facilmente obtidas por procedimentos não invasivos.

De nosso conhecimento, há apenas um estudo de revisão com enfoque nas características clínicas e genéticas da ST, mosaicismo, cromossomo Y e risco de tumor gonadal.^8^ Os dados apresentados nesse estudo apontaram: 1 – que a detecção de sequências do cromossomo Y em ST, independentemente do cariótipo, é necessária para prevenir o desenvolvimento de gonadoblastoma e 2 – a técnica de PCR deve ser empregada devido a alta sensibilidade, baixo custo e fácil execução.

Após a confirmação da ST pela citogenética, sugere-se a inclusão da técnica de PCR como complementação para detecção de sequências do cromossomo Y nessas pacientes. Essa técnica molecular é mais sensível e pode detectar a presença de material do cromossomo Y.

### Gonadoblastoma

O gonadoblastoma, frequentemente observado na segunda década de vida, é um tumor gonadal benigno com um alto potencial de transformação maligna.^4^ É uma lesão com bom prognóstico, pode diferenciar-se em tumor de células germinativas, como disgerminomas, e em menor frequência em teratomas, carcinoma embrionário, tumor de saco vitelínico e coriocarcinoma.^13^ Esse tumor está presente principalmente em mulheres com disgenesia gonadal e aproximadamente 95% delas têm material do cromossomo Y em seu genoma. Portanto, a detecção dessas sequências por técnicas citogenéticas e/ou moleculares tem sido estimulada para nortear a indicação profilática de cirurgia para retirada das gônadas nesse grupo de pacientes, uma vez que não são, em geral, tumores metastáticos e pela possibilidade de cura com a sua retirada.^4^ Mulheres com ST exibem disgenesia gonadal e, portanto, a detecção de sequências do cromossomo Y nessas pacientes é de extrema importância clínica.

A maior prevalência para gonadoblastoma nessa revisão foi 33%^16^^,^ 19 e está de acordo com outros estudos.^6^^,^ 27 Esse valor foi identificado em uma pesquisa recente e as quatro pacientes com ST tinham cariótipo 45,X/46,XY.^6^ Em duas delas, foi observado um disgerminoma (idade na cirurgia: 11,25 anos) e um teratoma (15 anos). Nas duas pacientes com gonadoblastoma apenas, a idade na cirurgia em anos foi 1,5 e 11,7.^6^ Outro estudo publicado recentemente relatou incidência de 35,3% de gonadoblastoma.^28^ Vinte pacientes com ST exibiram sequências do cromossomo Y de um total de 217 e 17 delas foram submetidas à gonadectomia.^28^ Uma frequência ainda maior foi relatada por Alvarez-Nava *et al*.,^29^ que investigaram 52 pacientes, detectaram quatro com sequências do cromossomo Y (7,7%), todas foram submetidas à gonadectomia e duas apresentaram gonadoblastoma (50%). Cinco estudos referiram incidências de 10% a 25%.^9^^,^ 11 ^-^ 13 ^,^ 18 Estudos prévios têm relatado frequências de 7%-10%^30^ e 16,7%.^31^ Esses achados evidenciam que a frequência de gonadoblastoma é variável e pode ser reflexo de uma investigação precoce.

Nos estudos conduzidos por Bianco *et al*.,^9^^-^ 12 os resultados obtidos foram: 1 – uma paciente 45,X com gonadoblastoma bilateral e amplificação positiva para o gene *SRY* nos três tecidos analisados;^9^ 2 – duas pacientes com gonadoblastoma bilateral [45,X: *SRY*+ e 45,X/46,X,r(?): *SRY* + *DYZ3* + ];^11^ 3 – duas pacientes com gonadoblastoma bilateral [45,X: *SRY*+ e 45,X/46,X,r(?): *SRY* +].^12^ Apesar de não ter sido identificado paciente com gonadoblastoma, o estudo histopatológico das gônadas de uma paciente revelou hiperplasia celular e luteoma estromal.^10^ Estudos posteriores também mostraram pacientes com esses achados histopatológicos.^11^^,^ 12

Outro gene associado à gonadoblastoma é o *OCT4* (*octamer-binding transcription factor* 4),^12^^,^ 13 também conhecido como *OCT3* ou *POU5FI*. Esse gene é considerado um marcador de tumores de células germinativas, tais como gonadoblastoma, disgerminoma, seminoma e outros, e sua expressão foi detectada por imuno-histoquímica em 100% dos casos de gonadoblastoma.^32^ O estudo publicado em 2010 mostrou alta expressão dos genes *SRY, TSPY* e *OCT4* em ambas as gônadas de uma paciente com resultado positivo por PCR (*SRY*+ e *TSPY* + ).^12^ Outro estudo revelou que a análise imuno-histoquímica para *OCT4* foi positiva em três casos (cariótipos: 45,X/46,XY: n=2 e 45,X/46,X,+mar: n=1), resultado sugestivo de tumor de células germinativas (gonadoblastoma ou carcinoma *in situ*).^13^ Nessa pesquisa, os 16 casos com sequências do Y foram submetidos à gonadectomia bilateral e as gônadas direita e esquerda avaliadas por coloração convencional com hematoxilina & eosina (H&E) e imuno-histoquímica para *OCT4*. Neoplasia gonadal não foi detectada em alguma das 32 gônadas avaliadas por H&E; entretanto, quatro gônadas (12%) de três pacientes (19%) exibiram positividade para *OCT4*, o que sugere a existência de tumores das células germinativas.^13^ Por esse motivo, esses autores recomendam um estudo histopatológico específico em gônadas, tais como imuno-histoquímica com *OCT4* para avaliar o risco real de desenvolvimento de tumores gonadais em pacientes com ST e sequências do cromossomo Y.^12^^,^ 13

Ainda em relação a pesquisas que relataram maior prevalência de gonadoblastoma, uma delas referiu duas pacientes com gonadoblastoma monolateral (cariótipos: 45,X e 45,X/46,X,+mar) e duas com gonadoblastoma bilateral (cariótipos: 45,X/46,XY)^16^ e outra, uma paciente 45,X/46,XY com gonadoblastoma.^19^ No estudo publicado em 2010, a cirurgia profilática foi feita em todas as nove pacientes com material do cromossomo Y antes dos 20 anos e uma delas, uma menina de cinco anos, a paciente mais jovem do estudo, exibiu gonadoblastoma bilateral sem qualquer sinal clínico.^18^ Essa paciente apresentou cariótipo 45,X/46,XY (GTG), wcpY + ^93^ (Fish) e positividade para todas as sequências específicas do Y (*SRY, TSPY1, DDX3Y* e *HSFY1*). A histologia de tecido ovariano não revelou gonadoblastoma em quatro das pacientes submetidas à gonadectomia.^20^

Um estudo histopatológico recente revelou tumores nas gônadas em seis de 11 pacientes (56%), incluindo quatro de sete (57%) com ST.^7^ Duas pacientes com ST exibiram gonadoblastoma nas gônadas direita e esquerda [cariótipos e idade na gonadectomia, respectivamente: 45,X/45,X,t(15;Y)(p11.2;q11.2) e dois anos e 11 meses; 45,X/46,XY e 10 anos e três meses]. Gonadoblastoma associado à disgerminoma foi observado em apenas uma gônada (direita em uma paciente e esquerda na outra) em outras duas pacientes (cariótipos 45,X/46,XY e 46,X,+der(15)/46,XY e idades: 11 anos e seis meses e 15 anos e quatro meses, respectivamente). A paciente mais jovem com ST submetida à gonadectomia tinha apenas dois anos.^7^

De acordo com os dados apresentados, pode-se concluir que é indicada a pesquisa molecular para sequências do cromossomo Y em pacientes com ST, independentemente do cariótipo, como complementação ao diagnóstico citogenético. A técnica sugerida é a PCR por ser de baixo custo, sensível, rápida e possibilitar o rastreamento de várias sequências do cromossomo Y simultaneamente. Também seria adequada a análise de um segundo tecido, além de sangue periférico. Naquelas pacientes com positividade para as sequências do cromossomo Y, é necessária a investigação de gonadoblastoma.
